# Ocular Ischemic Syndrome With Bilateral Carotid Artery Stenosis in a Patient With Chronic Tophaceous Gout

**DOI:** 10.7759/cureus.49270

**Published:** 2023-11-22

**Authors:** Amirah Mohammad Razali, Muhammad Adri Mohamed Shafit, Rafidah Md Saleh, Adzleen Mohmood, Mujammad Mohd Isa

**Affiliations:** 1 Department of Ophthalmology, Faculty of Medicine and Health Sciences, Universiti Putra Malaysia, Serdang, MYS; 2 Department of Ophthalmology, Hospital Sultan Abdul Aziz Shah, Universiti Putra Malaysia, Serdang, MYS

**Keywords:** smoking, metabolic syndrome, carotid artery stenosis, gouty arthritis, ocular ischaemic syndrome

## Abstract

Ocular ischemic syndrome is a rare, blinding condition that usually presents rather late. This occurs mainly due to stenosis or occlusion of the carotid artery which supplies the ocular structures. Bilateral involvement may occur in one in five cases but may be asymmetrical. We report a case of a 72-year-old gentleman with bilateral ocular ischemic syndrome secondary to left common carotid artery total occlusion and severe right proximal internal carotid artery stenosis in a patient who is an active smoker with chronic tophaceous gout. His vision remained stable after a year of follow-up, with the main emphasis on optimizing his medical condition and smoking cessation.

## Introduction

Ocular ischemic syndrome (OIS) is a rare disease with an estimated incidence of 7.5 cases per million persons per year [1]. The eye may be the only manifestation of severe systemic atherosclerosis in patients. The five-year mortality rate in OIS patients is up to 40%, with the majority of cases being due to cardiac disease, followed by cerebral infarction [1,2]. Symptoms of OIS may be non-specific which include blurring of vision, either sudden or gradual, visual field loss, and ocular or periocular pain. Ocular manifestation typically involves neovascularization of the iris or angles but may also occur at the optic disc and retina. Fundus features include pale optic disc or optic disc cupping, blot hemorrhages mainly in the mid-periphery, and vascular changes such as boxcarring of vessels, arteriolar attenuation, and central retinal vein occlusion [3]. As the clinical features are non-specific, this condition may be underdiagnosed. Common differentials are central retinal vein occlusion and diabetic retinopathy. Those with asymmetrical diabetic retinopathy should be screened for carotid artery stenosis as this is found in around 20% of these patients [4].

Atherosclerosis remains the main cause of OIS. This is contributed by various factors, mainly metabolic diseases which include diabetes, hypertension, and hyperlipidemia. The role of smoking has been extensively investigated. It is known to cause oxidative stress, vascular inflammation and dysfunction, platelet aggregation, and impairment of the serum lipid profile [5]. More recently, studies have shown that gout also contributes to carotid artery pathology. The monosodium urate crystal deposition contributes to subclinical inflammation, which then leads to vascular damage [6]. We report a case of bilateral OIS in a smoker with chronic tophaceous gout.

## Case presentation

**Figure 1:**
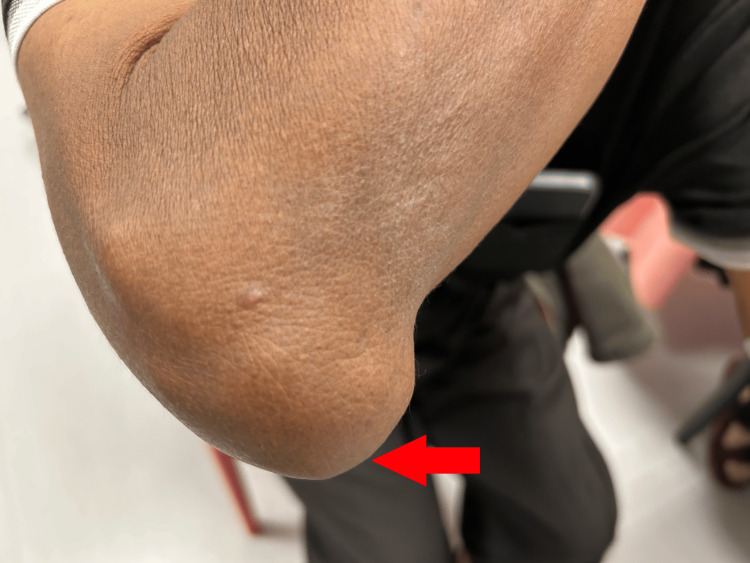
Right elbow with a large tophus (red arrow)

A 72-year-old gentleman with normal body mass index, who has underlying diabetes, hypertension, hyperlipidemia, chronic kidney disease, and chronic tophaceous gout (Figure 1), presented to the eye clinic with a one-month history of gradual, blurring of vision in the left eye. His diabetes and hypertension were well controlled with HBA1c of 6.1% and only required amlodipine 10 mg daily for high blood pressure.

The blurring of vision was associated with redness, eye pain and headache. He had a history of uncomplicated left eye cataract surgery five years ago. He is an active smoker with a 50-pack-year smoking history. On examination, his best corrected visual acuity was 6/6 in the right eye and 3/60 in the left eye. There was a positive relative afferent pupillary defect on the left eye. Anterior segment examination for the right eye was unremarkable except for a mildly cataractous lens with normal intraocular pressure. Left eye examination revealed injected conjunctiva, the cornea was clear, and the anterior chamber was deep and quiet. Rubeosis iridis was present with a slightly elevated IOP of 22 mmHg. All angles were closed on gonioscopy. Fundus examination of the right eye showed the presence of asteroid hyalosis with a pink optic disc and a cup-to-disc ratio of 0.5. There was no dot or blot hemorrhage seen. Left eye examination revealed a palish optic disc with a cup-to-disc ratio of 0.6, neovascularization seen at the disc, a few blot hemorrhages in the mid-periphery of the superior and inferior quadrant with veins which were slightly dilated but not tortuous.

Optical coherence tomography (OCT) macula of the right eye was normal while OCT macula of the left eye revealed inner retinal layer thinning. He was initially treated for left-eye neovascular glaucoma secondary to an old ischemic central retinal vein occlusion. In view of the atypical presentation, a carotid Doppler was arranged to rule out OIS. Carotid doppler showed long segment stenosis of the left common carotid artery and bilateral internal carotid arteries with underlying features of atherosclerotic disease. We proceeded with cerebral angiogram which revealed total occlusion of the left common carotid artery with established collateral supply. There was also severe short segment stenosis of the right proximal internal carotid artery with up to 90% occlusion. The left vertebral artery was also severely stenosed but with the presence of established collaterals. The right vertebral artery was normal. MRI brain showed only small vessels disease and no area of infarction.

**Figure 2:**
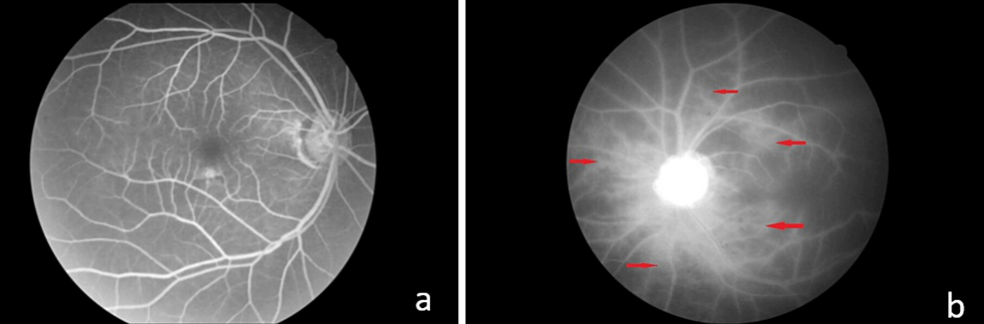
Fundus fluorescein angiogram of the right eye at 1 min 30 seconds (a) and the left eye at 3 minutes (b) showing multiple hypo-perfused area over the left retina with staining along the retinal vessels resembling frosted branch angiitis (red arrows).

Fundus fluorescein angiogram was done. For the right eye, there was prolonged choroidal filling time and prolonged retinal arteriovenous time. These was no area of capillary non-perfusion. For the left eye, there was delayed in both choroidal and retinal circulation with multiple hypo-perfused areas and staining along all the retinal vessels resembling frosted branch angiitis (Figures 2a, 2b).

Blood investigations including full blood count, electrolytes, fasting blood sugar and liver function test were all normal. The HbA1c was 6.1%. The urea was slightly elevated at 9.7mmol/L with creatinine of 129µmol/L and eGFR of 44.1 mL/min/1.73m2. Uric acid was elevated at 538µmol/L. Cholesterol level was normal 4.5mmol/L but with slightly elevated triglyceride at 3.6mmol/L, LDL of 1.9 and HDL of 1mmol/L. ESR was 22mm/hr and CRP 11.

A final diagnosis of bilateral OIS with left eye neovascular glaucoma was made. Patient was treated with pan-retinal photocoagulation over the left eye. He was also started on timolol twice daily to lower down the intraocular pressure together with dexamethasone and atropine eyedrops.

For the left complete carotid artery stenosis, the decision was not for surgical intervention in view of the established collaterals. For the right common carotid artery with 90% stenosis, multiple extensive discussions were made with the neurologist, intervention radiologist and rheumatologist, together with patient and family for possible stenting. Since the patient was not keen on surgical intervention, in particular the risk of stroke, he was started on a single antiplatelet therapy and optimization of his medical condition. He was also reviewed by the cardiologist and there was no evidence of ischemic heart disease based on normal electrocardiogram and echocardiogram. He was also strongly advised for smoking cessation.

**Figure 3:**
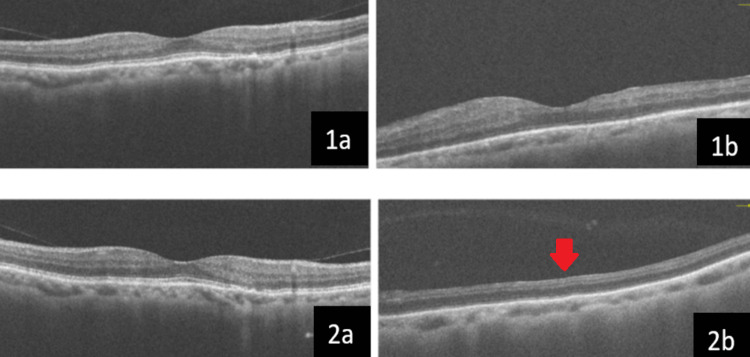
OCT macula of the patient. At presentation (1a, 1b) and after one year of follow-up (2a, 2b). The right eye (1a) had normal foveal contour and retinal layers with slight disruption of the ellipsoid zone at the parafoveal area. There were areas of inner retinal layer thinning for the left eye (1b). The right eye (2a) remained the same while for the left eye (2b), there was macula atrophy with loss of the foveal contour (red arrow).

His vision has maintained 6/6 over the right eye over the past 12 months, with an IOP of 10mmHg and no neovascularization. For the left eye, the vision was counting finger with normal IOP of 14mmHg and despite complete pan-retinal photocoagulation, there was minimal remaining rubeosis present. The OCT macula of the right eye showed normal foveal contour with a foveal avascular zone of 368µm, an increase from 250µm from the initial presentation based on OCT angiography. The left eye shows macula atrophy (Figure 3).

## Discussion

OIS is a rare, blinding condition in which ocular hypoperfusion occurs due to severe carotid artery occlusion or stenosis. The bilateral OIS may occur in up to 22% of cases [3]. The main cause of OIS is atherosclerosis with other causes including giant cell arteritis, Takayasu arteritis, and fibrovascular dysplasia [1]. Since the main cause of OIS is atherosclerosis, the majority of the patients have other metabolic diseases which include hypertension in 73% and diabetes in 56% [2]. There has been a recent discussion on the role of uric acid crystals in promoting inflammation. A study by Hammer et al. found that urate crystal deposition contributes to subclinical inflammation leading to carotid artery stenosis, with large tophus being more associated with carotid plaque in view of the chronicity [6]. This is also what we observe in our patient with chronic tophaceous gout who has slightly elevated ESR and CRP together with extensive vascular narrowing and occlusion.

Based on a systematic review by Terelak-Borys et al., blurring of vision is the most common symptom of OIS, which is present in over 90% of patients [1]. This may occur gradually over weeks or months, and it may also occur suddenly due to central retinal artery occlusion. Other symptoms are visual field defects and ocular or periocular pain (40%) [1]. Up to 21% of the patients may be asymptomatic [3]. The commonest anterior segment manifestations of OIS are iris neovascularization (87%) followed by neovascularization at the angle (59%) [3]. Others include conjunctival hyperemia, cornea edema, peripheral anterior synechiae, atrophic sphincter pupillae, semi-dilated pupil, asymmetrical cataracts, and scleral melting [1]. For posterior segment manifestation, the commonest finding at presentation is a pale optic disc (28%), followed by superficial retinal hemorrhages (23%) and retinal arteriolar attenuation (21%) [3]. Other manifestations include optic disc cupping, optic disc edema, anterior and posterior ischemic optic neuropathy, optic disc neovascularization, retinal neovascularization, dilated retinal veins, spontaneous retinal arteries pulsation, microaneurysms, cherry-red spot, cholesterol emboli, cotton wool spots, vitreous hemorrhage and areas of chorio-retinal atrophy [1,3].

Gouty arthritis is the commonest inflammatory arthritis in men with a prevalence of up to 6.8% depending on population with an incidence of 0.58 to 2.89 per 1,000 person-years [7]. Clinically, there are four stages of gout which are asymptomatic hyperuricemia, acute gouty arthritis, intercritical gout, and chronic tophaceous gout [8]. Gout is known to increase the risk of cardiovascular disease and chronic kidney disease. Hammer et al. found that in patients with intercritical gout, uric crystal deposition contributes to subclinical inflammation and vascular complication as shown by higher calprotectin levels in those with large tophi with more frequent carotid plaque [6].

Gout may also affect the eye with ocular manifestations of uric acid crystal deposition including periocular, sclera, cornea and iris tophi deposition, episcleritis, cortical cataract, retinal vascular occlusion, and asteroid hyalosis [9]. Lu et al. found that uric acid levels and their fluctuation affect the retinal and choroidal microcirculation with greater serum uric acid levels being associated with lower superficial vessel density and choriocapillaris flow deficit [10]. Yang et al also found similar results mainly in men and highlighted that those with higher serum uric acid levels are more likely to develop ocular complications and visual impairment [11]. In patients with underlying metabolic disease and chronic gout, carotid artery stenosis may occur more frequently than expected owing to the subclinical inflammation and vascular occlusion that uric crystals may cause [6,9,12]. When combining the macrovascular complications of gout with its microvascular complications to the eye, it may cause devastating blindness due to OIS as both the macrovascular and microvascular supply may be affected. Since clinical features of OIS can be non-specific, in patients with metabolic disease and gout, a carotid Doppler is strongly suggested to look for carotid artery stenosis.

The general management of OIS is mainly to treat the underlying carotid stenosis and restore the ocular perfusion. Options include carotid artery endarterectomy, carotid artery stenting, and rarely extracranial-intracranial arterial bypass surgery [1,3]. This depends on various factors including comorbidities and the extent of the stenosis. It is also paramount to address any underlying metabolic risk factors such as hypertension, diabetes, hyperlipidemia, and obesity [1]. Optimization of the uric acid levels is also vital as the subclinical inflammation induced by the urate crystals may cause further worsening of vascular stenosis [12]. Smoking cessation is important as smoking causes thrombus formation in atherosclerotic arteries. There was a dose-response relationship between cigarette smoking and the occurrence of extracranial carotid atherosclerotic stenosis, with one cigarette smoked per day increasing the risk by 1% [13]. It was also found that smoking and dyslipidemia were more associated with extracranial atherosclerotic stenosis in males [14].

Ocular management involves managing complications such as neovascular glaucoma. This occurs due to chronic retinal and choroidal ischemia, leading to neovascularization which occurs mainly at the anterior segment but may also involve the posterior segment of the eye. Pan-retinal photocoagulation is effective in around 36% of patients as the ischemic drive from the choroidal ischaemic is not halted [1]. Anti-VEGF treatment has also been used to treat OIS but this requires serial injections in which the disease recurred every time the injections were stopped [15]. Topical antiglaucoma may be useful at the initial stage, but later on, patients may need glaucoma filtration surgery or tube surgery to control intraocular pressure. Normal tension glaucoma may also occur in OIS due to reduced ocular perfusion, hence a compromised blood supply of the optic nerve head.

## Conclusions

Poor control of systemic disease may have devastating ocular complications leading to blindness. There is a need for optimization of uric acid levels as it can also cause macrovascular and microvascular complications which may potentiate pre-existing vascular disease. Smoking remains one of the important modifiable risk factors for atherosclerosis. A multidisciplinary approach is vital in managing patients with OIS, which may present initially to an ophthalmologist to ensure a good outcome for patients.
